# Proliferative retinopathy in nail-patella syndrome

**DOI:** 10.1016/j.ajoc.2025.102320

**Published:** 2025-04-08

**Authors:** Liangbo Linus Shen, Amelia Bhisitkul, Gregory M. Lewis, Sidhiporn Borirakchanyavat, Robert B. Bhisitkul

**Affiliations:** a Department of Ophthalmology, University of California, San Francisco, Wayne and Gladys Valley Center for Vision, 490 Illinois Street, 54H, San Francisco, CA, USA; b Department of Ophthalmology, Kaiser Permanente Medical Group, San Francisco, CA, USA

## Abstract

**Purpose:**

Nail-Patella Syndrome (NPS) is a rare autosomal dominant disease. Associated ocular abnormalities in NPS are well established, involving the anterior segment and predisposition to open-angle glaucoma. We report a patient with NPS who presented with bilateral proliferative retinopathy and cystoid macular edema (CME).

**Observations:**

A 45-year-old male with genetically confirmed NPS presented with typical systemic features of NPS, including dystrophic nails, hypoplastic patellae, and limited elbow extension. He had vision loss associated with optic disc neovascularization (NVD), vitreous hemorrhage, and CME in both eyes. Systemic work-up ruled out common causes of proliferative retinopathy. Treatment with intravitreal bevacizumab and subsequent aflibercept injections partially improved the retinal pathology.

**Conclusion and importance:**

This case documents retinal abnormalities, specifically proliferative retinopathy and CME, in a patient with NPS. These findings suggest that retinal pathology might be an aspect of NPS, highlighting the importance of fundus exams and retinal imaging for patients with NPS who present with visual symptoms.

## 1 Introduction

Nail-Patella Syndrome (NPS) is an autosomal dominant genetic disease resulting from variants in the homeobox transcription factor *LMX1B* gene on chromosome 9q34, affecting about 1 in every 50,000 people worldwide.^1^ NPS is a pleiotropic condition that can manifest as a range of phenotypic abnormalities in the skeletal system, the kidneys, and the eyes. Characteristic skeletal features include dystrophic fingernails and toenails, absent or hypoplastic patellae, prone to dislocation, and early degenerative arthritis. Other skeletal abnormalities include iliac horns, congenital hip dislocation, and various joint, foot, and spine problems. Renal manifestations usually present with proteinuria, the effects of which can range from asymptomatic to chronic renal failure, leading to early mortality. Intestinal issues can include irritable bowel syndrome and diverticulitis.

Ocular manifestations in NPS usually include glaucoma and anterior segment dysgenesis. Open-angle glaucoma (OAG) and ocular hypertension are the most common ocular associations, for which early screening is recommended for NPS patients.^1^^,^^2^ - *LMX1B* encodes a transcription factor involved in the embryonic development of the cornea and ciliary body. Anterior segment findings can include abnormal pigmentation of the inner margin of the iris (Lester's sign), pupillary ectopia, sclerocornea, microcornea, keratoconus, congenital cataract, and microphakia. Ptosis, epicanthal folds, and hypertelorism can be present. No retinal findings have been associated with NPS, but focal areas of reduced retinal vascular density in the perifoveal and peripapillary regions on OCT-angiography have been reported in an NPS with OAG.^3^ We present here a case of a 45-year-old man with NPS who developed proliferative retinopathy with bilateral NVD, vitreous hemorrhage, and cystoid macular edema (CME).

## 2 Case report

**Fig. 1:**
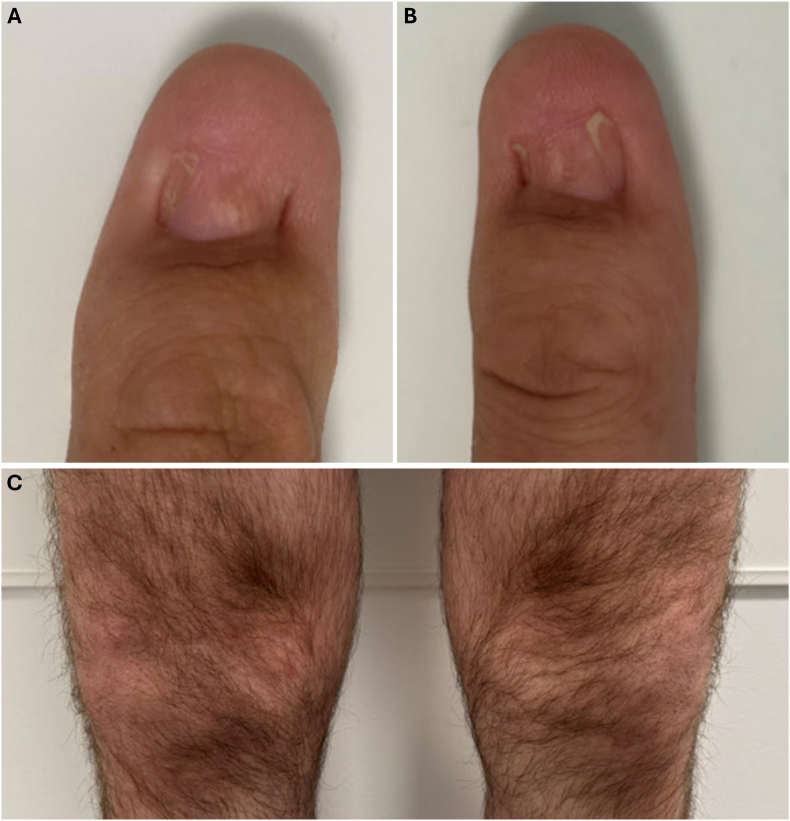
Systemic manifestations of patient with nail-patella syndrome. **A**, **B**, Hypoplastic thumb-nails of the both hands. **C**, Absent patella in the right knee and small patella in the left knee.

A 45-year-old Caucasian male who was diagnosed with Nail-Patella Syndrome (NPS) at age 3 years was referred to the retina service at the University of California San Francisco (UCSF) for bilateral proliferative retinopathy. Genetic testing of variants in the *LMX1B* gene was done by next-generation sequencing by Invitae (Invitae Corporation, San Francisco, USA; CLIA certified laboratory). The testing was positive for a pathogenic, heterozygous variant c.679_680del (p.Thr227Hisfs∗28) in the Exon 4 of the *LMX1B* gene, that was previously reported in the ClinVar database (ClinVar Variation ID: 2854672) as pathogenic in NPS (autosomal dominant inheritance).^1^ He does not have a family history of NPS. The patient had several systemic findings and symptoms of NPS, including hypoplastic thumbnails in both hands (Fig. 1A and B), split nails in the index figure of both hands, absent patella in the right knee (Fig. 1C), small patella in the left knee (Fig. 1C), and limited extension of the right elbow with restriction of supination. The patient had no history of diabetes or hypertension.

The patient reported several months’ history of intermittent bilateral blurry vision in both eyes. Seven months prior, he was diagnosed with bilateral retinal edema and treated with topical prednisolone acetate twice daily in both eyes but developed elevated intraocular pressure (IOP) to 34 mmHg in the left eye. Consequently, prednisolone acetate was tapered, and timolol was prescribed twice daily for the left eye, normalizing the IOP. Subsequently, he was referred to a retina specialist who diagnosed him with neovascularization of the optic disc (NVD) and intraretinal fluid in both eyes. Systemic work-up was noncontributory, including hemoglobin A1C of 5.1, normal urinalysis, unremarkable carotid ultrasound, and negative serologic testing (complete blood count, basic metabolic panel, alanine transaminase levels, thyroid-stimulating hormone levels, lipid panel, hemoglobin electrophoresis, treponema pallidum particle agglutination, quantiferon gold, HIV 1 and 2 nucleic acid test, angiotensin-converting enzyme levels, lysozyme levels). Treatment was initiated with one dose of intravitreal bevacizumab injections in both eyes four months prior. One month prior, he developed anterior chamber inflammation with 2+ anterior chamber cells in the right eye and trace anterior chamber cells in the left eye, without signs of vitritis or posterior uveitis; the iritis resolved with prednisolone acetate four times daily and timolol twice daily in both eyes.

**Fig. 2:**
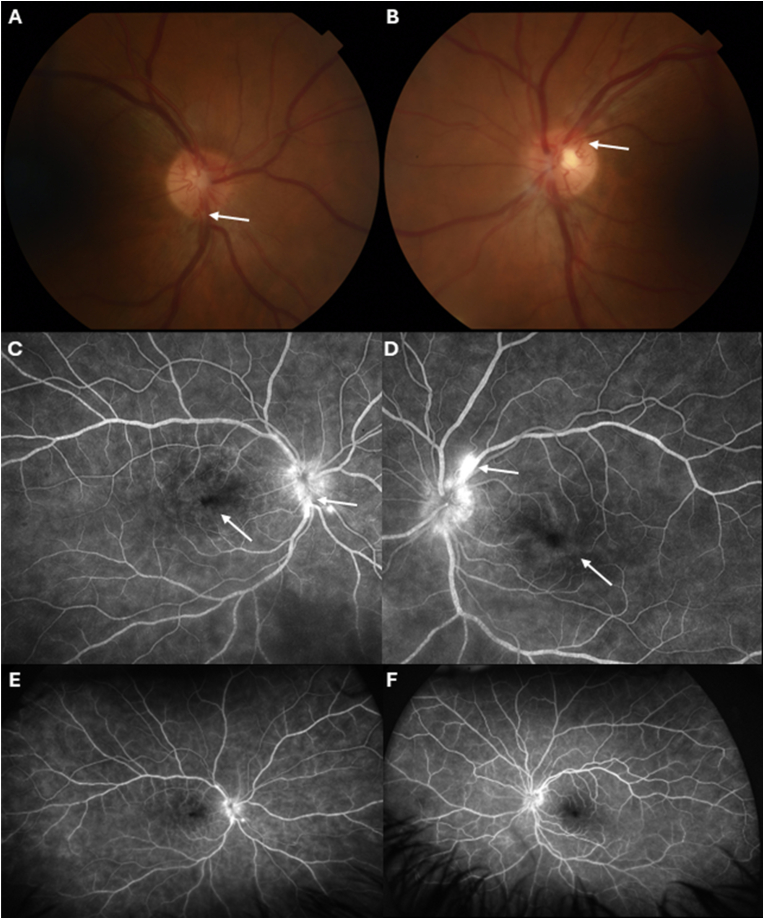
Retinal imaging of patient with nail-patella syndrome at presentation. **A and B**, Color photographs of the optic disc showing the neovascularization (arrows) at the optic disc of the right (**A**) and left (**B**) eyes. **C and D**, Fluorescein angiography of the right (**C**) and left (**D**) eyes demonstrating bilateral neovascularization (arrows) at the optic disc and leakage in the macula as well as cystoid macular edema (arrows) and patchy choroidal staining without significant capillary dropout. **E and F**, Wide-field fluorescein angiogram of the right (**E**) and left (**F**) eyes, demonstrating no significant capillary dropout or peripheral nonperfusion in either eye.

**Fig. 3:**
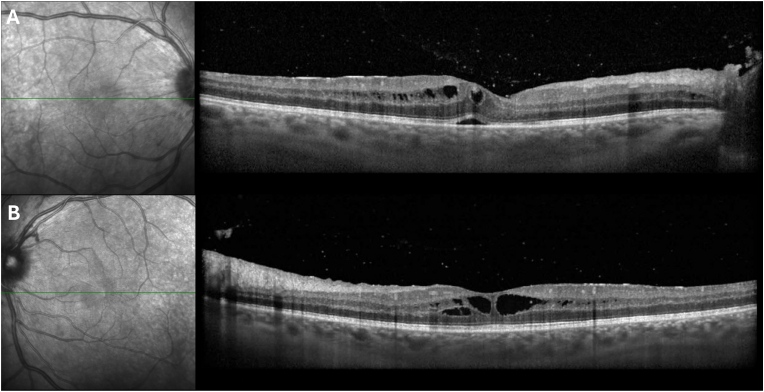
Optical coherence tomography of the right (**A**) and left (**B**) eyes in the patient with nail-patella syndrome at presentation demonstrating cystoid macular edema.

At his initial UCSF visit, the patient's visual acuity was 20/30 in the right eye and 20/25 in the left eye. IOP was 14 mmHg in the right and 10 mmHg in the left eye. Slit-lamp examination revealed no significant anterior segment findings, with no cell or flare in the anterior chamber or anterior vitreous of both eyes. Fundus examination of the right eye revealed a cluster of cotton wool spots in the superior peripheral retina and neovascularization at the optic disc (Fig. 2A). The left eye showed a small cotton wool spot infranasal to the optic disc and neovascularization at the optic disc (Fig. 2B). Fluorescein angiography (Fig. 2C–F) showed neovascularization of the disc, cystoid macular edema (CME), and patchy filling of choroidal circulation in both eyes. Arteriovenous filling was normal in the left eye, and there was no significant capillary non-perfusion or vascular dropout, nor signs of retinal vasculitis in either eye. Optical coherence tomography (OCT; Fig. 3A and B) showed CME in both eyes with some subretinal fluid in the right eye. Intravitreal bevacizumab was administered in both eyes during this visit.

Over the ensuing months, intravitreal bevacizumab was switched to aflibercept injections in both eyes at 6 weeks, 9 weeks, and 5 months post-presentation due to the persistent macular edema unresponsive to the initial bevacizumab injections. Seven months after the initial UCSF visit, the patient's visual acuity was 20/30 in both eyes. IOP was 11 mmHg in the right eye and 19 mmHg in the left eye. A slit-lamp examination showed no cell or flare in the anterior chamber or anterior vitreous area of either eye. Fundus examination of the right eye showed fibrosed neovascularization at the disc, a small inferior vitreous hemorrhage, and a prominent vitreous band from released hyaloid. The left eye had fibrosed neovascularization at the disc and posterior vitreous detachment with vitreous debris. OCT showed improved CME in both eyes with resolved subretinal fluid in the right eye.

## 3 Discussion

While this 45-year-old man with NPS exhibited classic systemic features, including hypoplastic nails, absent and hypoplastic patellae, and limited elbow extension, his ocular findings of bilateral proliferative retinopathy and macular edema are not usually associated with NPS. Ocular abnormalities in NPS have been widely reported, being limited to the anterior segment, including a range of open-angle glaucomas^2^^,^^4^^,^^5^ and corneal, iris and lens abnormalities.^4^ Anterior segment dysgenesis can be explained by the NPS variants in the *LMX1B* gene, which encodes a LIM-homeodomain transcription factor critical for the development of the limbs, kidneys, and the anterior segment of the eye.^6^^,^^7^ Animal studies have demonstrated that disruptions in the *LMX1B* gene can result in abnormal development of trabecular meshwork, elevated IOP, and glaucomatous changes in mice.^8^^,^^9^

The mechanism by which NPS leads to retinal pathology is unclear. One possibility is that disruptions in the *LMX1B* gene could result in abnormal vascular development, leading to retinal ischemia and subsequent neovascularization. Animal studies have demonstrated that inactivation of the *LMX1B* gene can result in cornea neovascularization, supporting the theory of abnormal vascular development linked to *LMX1B* dysfunction.^8^ A previous case report has shown focal areas of reduced vessel density in the macular and circumpapillary regions of a patient with NPS on OCT angiography^3^; however, this was suggested to be related to the patient's glaucoma status rather than a developmental defect in the retinal vasculature. Additionally, internal carotid artery aplasia has been reported in a patient with NPS, which indicates that cerebrovascular developmental abnormalities may be associated with the syndrome^10^ and could offer an alternative explanation of proliferative retinopathy in this patient's case arising from ocular ischemic syndrome. But carotid Doppler studies performed on our patient were normal. An alternative mechanism for retinal neovascularization is suggested by animal studies showing that variants in *LMX1B* affect collagen expression in the glomerular basement membrane,^11^ likely contributing to the renal pathology and nephrosis in NPS. A similar impact on retinal collagen could contribute to vascular compromise and neovascularization observed in our patient with NPS.

We cannot rule out the possibility that the observed retinal pathology is coincidental in our patient with NPS. For example, the episode of bilateral anterior chamber inflammation together with the presence of CME could indicate a uveitic etiology or an undetected systemic inflammatory process. Other common causes of proliferative retinopathy, such as diabetes, hypertension, or retinal vascular occlusion, were absent in this patient, and he was on no significant medications known to cause CME. An understanding of the retinal effects of NPS awaits further cases and research to elucidate pathogenic mechanisms. Nevertheless, the occurrence of bilateral proliferative retinopathy with chronic CME in a working-age patient raises the possibility that retinal disorders are part of the range of ocular abnormalities associated with NPS, and that in addition to glaucoma evaluation, screening with fundus exam and macular OCT is merited in these patients.

## 4 Conclusions

This case describes retinal abnormalities, including CME and retinal neovascularization, in a patient with NPS. These retinal findings expand the spectrum of ocular manifestations associated with NPS, although the underlying mechanisms remain unclear. The presence of retinal pathology should be considered in NPS patients who present with visual symptoms.

## CRediT authorship contribution statement

**Liangbo Linus Shen:** Writing – review & editing, Writing – original draft, Methodology, Investigation, Data curation, Conceptualization. **Amelia Bhisitkul:** Writing – review & editing, Writing – original draft, Data curation, Conceptualization. **Gregory M. Lewis:** Writing – review & editing, Data curation, Conceptualization. **Sidhiporn Borirakchanyavat:** Writing – review & editing, Data curation, Conceptualization. **Robert B. Bhisitkul:** Writing – review & editing, Writing – original draft, Supervision, Resources, Methodology, Investigation, Funding acquisition, Data curation, Conceptualization.

## Claims of priority

After conducting a literature review on 07/10/2024 utilizing PubMed, and Google Scholar, using the key words ("nail-patella syndrome", "Hereditary onycho-osteodysplasia", "Fong disease", "Hereditary osteo-onychodysplasia"), we did not find any prior reports of proliferative retinopathy in patients with nail-patella syndrome.

## Patient consent

Consent to publish this case has been obtained from the patient in writing. This case does not contain any personal identifying information.

## Authorship

All authors attest that they meet the current ICMJE criteria for authorship.

## Funding sources

This work was supported by the UCSF Vision Core shared resource of the NIH/NEI P30 EY002162 and the All May See Foundation.

## Declaration of competing interest

The authors declare that they have no known competing financial interests or personal relationships that could have appeared to influence the work reported in this paper.
